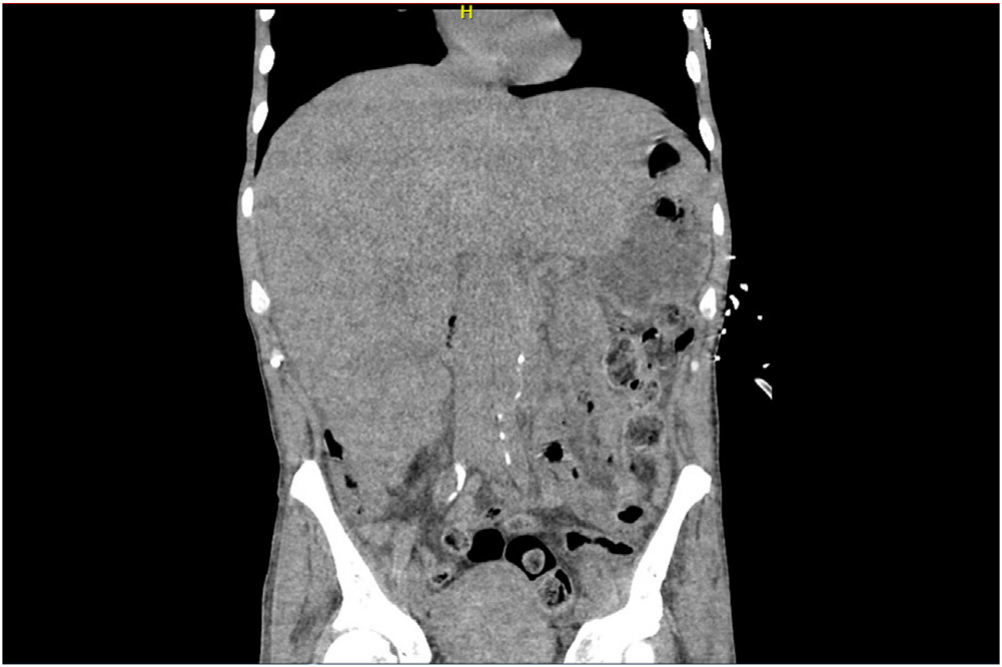# Metastatic Prostate Cancer Presenting As Advanced Hepatic Failure

**DOI:** 10.1016/j.gastha.2023.08.006

**Published:** 2023-08-19

**Authors:** Lisette Collins, Jamie O. Yang, Michael E. Lazarus

**Affiliations:** 1 Department of Internal Medicine, David Geffen School of Medicine at University of California, Los Angeles, Los Angeles, California; 2 Department of Internal Medicine, University of California, Los Angeles, Los Angeles, California

Fifty-nine-year-old male with a history significant for prostate adenocarcinoma complicated by metastasis to liver, bone, lung, and inguinal nodes being treated with Lupron and darolutamide who presented to the emergency department with intractable nausea, vomiting, abdominal pain, and confusion. Abdominal exam notable for right upper quadrant rigidity and epigastric tenderness to palpation. On admission, his labs were notable for aspartate aminotransferase 713 IU/L, alanine transaminase IU/L 282, alkaline phosphatase 968 IU/L, total bilirubin 4.6 micromoles/L, uric acid 18.8 mg/dL, lactate dehydrogenase 1344 U/L, and lactate 5.83 mmol/L. Computerized tomography abdomen/pelvis demonstrate an enlarged liver measuring 25 cm with innumerable hepatic masses nearly replacing the hepatic parenchyma (Figure). Given the elevated uric acid level and advanced clinical presentation, there was concern for the transformation of his tumor into a more aggressive variant. He underwent a liver biopsy which did not show evidence of neuroendocrine small cell carcinoma and was consistent with metastatic prostatic adenocarcinoma. He was ultimately diagnosed with fulminant liver failure secondary to metastatic prostate adenocarcinoma. His hospital course was complicated by rising bilirubin levels, coagulopathy, encephalopathy, and acute renal failure. Given the overall poor prognosis and worsening condition, the patient was placed on comfort care and died on day 9 of hospital admission.

**Figure:**